# Bone Defect Regeneration and Donor-Site Morbidity After Bone–Patellar Tendon–Bone Anterior Cruciate Ligament Reconstruction: A Prospective Cohort Study

**DOI:** 10.3390/medicina62061203

**Published:** 2026-06-22

**Authors:** Milan Milinkov, Oliver Dulić, Mile Bjelobrk, Milan Tošić, Branko Baljak, Mihail Mirković, Mirko Obradović

**Affiliations:** 1 Clinic for Orthopedic Surgery and Traumatology, University Clinical Center of Vojvodina, 21000 Novi Sad, Serbia; oliver.dulic@mf.uns.ac.rs (O.D.); mile.bjelobrk@mf.uns.ac.rs (M.B.); milan.tosic@mf.uns.ac.rs (M.T.); branko.baljak@mf.uns.ac.rs (B.B.); mirko.obradovic@mf.uns.ac.rs (M.O.); 2 Department of Surgery, Faculty of Medicine, University of Novi Sad, 21137 Novi Sad, Serbia; 3 Faculty of Medicine, University of Novi Sad, 21137 Novi Sad, Serbia; mirkovicmihail@gmail.com

**Keywords:** anterior cruciate ligament, bone–patellar tendon–bone graft, donor-site morbidity, kneeling pain, magnetic resonance imaging

## Abstract

*Background and Objectives*: This prospective cohort study aimed to assess patellar and tibial donor-site bone defect volume and regeneration on MRI at 4 weeks and 12 months after bone–patellar tendon–bone anterior cruciate ligament reconstruction and to determine their association with knee function and donor-site morbidity at 12 months. *Materials and Methods*: This single-center prospective observational cohort study included 30 patients who underwent ACL reconstruction with a BTB autograft. Donor-site bone defect volume was estimated on MRI using a triangular prism approximation at 4 weeks and 12 months by two independent evaluators blinded to patient-reported outcome scores. Clinical outcomes were assessed at 12 months using the International Knee Documentation Committee (IKDC) subjective knee form and the Donor Site Morbidity Questionnaire (DSMQ). Associations between MRI-derived parameters and patient-reported outcomes were analyzed using Spearman’s rank correlation coefficient. *Results*: At 4 weeks, mean donor-site bone defect volume was 2602.4 ± 684.7 mm^3^ in the patella and 2993.9 ± 714.3 mm^3^ in the tibia. At 12 months, defect volume decreased to 628.0 ± 279.7 mm^3^ and 980.8 ± 488.2 mm^3^, respectively. Tibial defects were significantly larger than patellar defects at both time points, while regeneration was significantly greater in the patella than in the tibia (74.8 ± 11.5% vs. 67.2 ± 15.1%; *p* = 0.0264). Regeneration was not significantly associated with IKDC or DSMQ scores (all *p* > 0.05). Larger patellar defect volume at 4 weeks was associated with worse subjective outcomes (both *p* = 0.0107). *Conclusions*: After BTB ACL reconstruction, tibial donor-site bone defects were larger, whereas patellar defects showed greater regeneration over time. Larger patellar defect volume at 4 weeks, but not regeneration percentage, was associated with worse subjective outcomes at 12 months.

## 1. Introduction

Anterior cruciate ligament (ACL) reconstruction is currently one of the most commonly performed procedures in orthopedic surgery [1]. This procedure is considered a standard treatment option for patients with functional knee instability, as it restores joint stability and reduces the risk of further articular cartilage damage [2]. Among the various reconstruction techniques, the bone–patellar tendon–bone (BTB) autograft has been described as the gold standard due to its biomechanical advantages, including bone-to-bone integration, improved rotational knee stability, reliable fixation, and a faster return to sports activities [2,3].

Despite its advantages, BTB autograft is associated with specific donor-site complications, which are described in the literature as donor-site morbidity. The most common manifestations include anterior knee pain, local tenderness, sensory disturbances in the anterior aspect of the knee, as well as kneeling difficulty [4]. These symptoms are of particular importance in young and active individuals, in whom the ability to kneel may represent an essential function due to religious, cultural, sporting, and occupational activities [5].

According to the available data, anterior knee pain after anterior cruciate ligament reconstruction using a BTB autograft may be present in as many as 62.9% of patients three months postoperatively, with a 1.7-fold higher likelihood of occurrence compared with patients in whom the semitendinosus tendon was used as a graft [6]. The formation of patellar and tibial bone defects during graft harvesting, as well as potential injury to the infrapatellar branch of the saphenous nerve, is considered to contribute to the development of these symptoms [7,8].

The clinical relevance of donor-site morbidity is further illustrated by reports that 51% of patients are unable to kneel because of pain after surgery, while 42.2% report numbness one year after reconstruction. These symptoms may be particularly limiting in activities that require substantial knee support, such as wrestling, martial arts, and rhythmic gymnastics [9].

MRI-based quantitative assessment may provide a more objective evaluation of donor-site bone healing than purely descriptive imaging findings. Such an approach may help characterize the extent of structural regeneration over time and may improve understanding of whether radiological healing is related to postoperative symptoms and functional recovery [10]. Patellar and tibial donor-site defects may also exhibit different healing patterns because of differences in local anatomy, biomechanical loading, and biological healing conditions.

Previous MRI studies of donor-site healing after BTB graft harvest have mainly focused on patellar tendon healing rather than on quantitative assessment of osseous donor-site regeneration [11,12]. To our knowledge, no previous study has quantitatively evaluated both patellar and tibial donor-site bone defects on MRI and examined their association with patient-reported outcomes after BTB ACL reconstruction.

This study aimed to assess patellar and tibial donor-site bone defect volume and regeneration on MRI at 4 weeks and 12 months after BTB ACL reconstruction and to determine their association with knee function and donor-site morbidity at 12 months.

We hypothesized that patellar and tibial donor-site defects would show different patterns of regeneration over time and that less favorable donor-site healing would be associated with worse patient-reported outcomes at 12 months.

## 2. Materials and Methods

### 2.1. Study Design and Patients

This single-center prospective observational cohort study was conducted at the Clinic for Orthopedic Surgery and Traumatology, University Clinical Center of Vojvodina, in patients undergoing anterior cruciate ligament reconstruction with a BTB autograft. Consecutive eligible patients were recruited from 1 November 2022 to 28 February 2023. During the recruitment period, 30 consecutive eligible patients aged 20 to 45 years were identified, and all were included in the study. Demographic data were obtained from the institutional clinical information system. Inclusion criteria were age between 20 and 45 years, primary ACL reconstruction using a BTB autograft, and availability of complete clinical and radiological follow-up data. Exclusion criteria were previous surgery on the same knee, revision ACL reconstruction, multiligament knee injuries, concomitant meniscal injuries requiring surgical treatment, significant chondral lesions, infection, fractures, knee tumors, or incomplete radiological documentation. Generalized joint laxity was not systematically recorded and was therefore not analyzed as a study variable. Previous surgery on the contralateral knee was not considered an exclusion criterion. No patients included in the study were lost to follow-up. Smoking status, activity level, and sports participation were not systematically recorded and were therefore not included in the analysis.

### 2.2. Surgical Technique

All procedures were performed arthroscopically by the same surgeon using a standardized surgical technique. A BTB autograft was harvested from the central third of the patellar tendon, together with bone blocks from the patellar and tibial insertions. The graft was prepared in a standardized fashion and used for anterior cruciate ligament reconstruction [3]. The osseous part of the graft was standardized to 25 mm in length and 10 mm in diameter. The donor-site bone defects created at the patella and tibia during graft harvesting were left unfilled and were not augmented with bone graft or synthetic material. Femoral and tibial bone tunnels were created arthroscopically, and graft fixation was performed using interference screws. All patients underwent postoperative rehabilitation according to a modified Shelbourne protocol, including early range-of-motion exercises, progressive weight-bearing, and staged strengthening [13]. Postoperative analgesic and anti-inflammatory therapy was prescribed by the attending anesthesiologist according to routine clinical practice and was not analyzed as a study variable.

### 2.3. MRI Assessment

**Figure 1:**
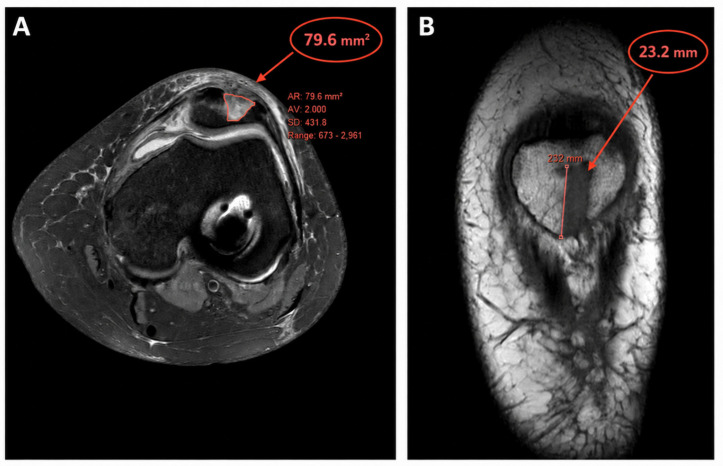
Patellar donor-site bone defect 4 weeks after anterior cruciate ligament reconstruction with a bone–patellar tendon–bone autograft: (**A**) axial AX PD SPAIR MRI showing the defect area used for base estimation; (**B**) coronal T1 TSE MRI showing the longitudinal extent of the defect.

**Figure 2:**
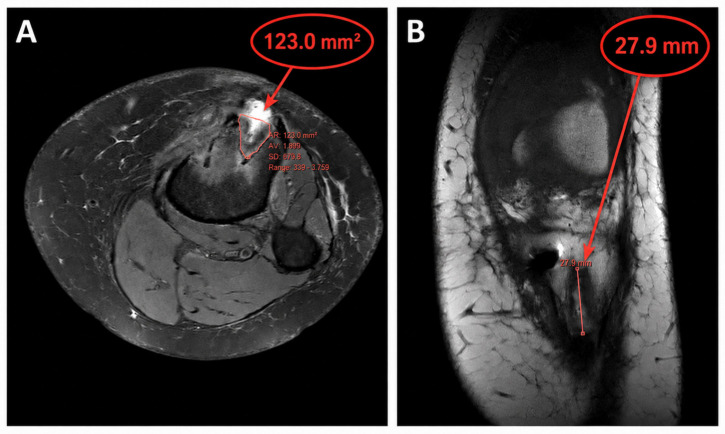
Tibial donor-site bone defect 4 weeks after anterior cruciate ligament reconstruction with a bone–patellar tendon–bone autograft: (**A**) axial AX PD SPAIR MRI showing the defect area used for base estimation; (**B**) coronal T1 TSE MRI showing the longitudinal extent of the defect.

Magnetic resonance imaging of the operated knee was performed at the Center for Radiology, University Clinical Center of Vojvodina, using a Philips Ingenia 3.0 T scanner (Philips Healthcare, Best, The Netherlands). Bone defect volume was estimated using MRI-derived measurements obtained in two planes. The center and longitudinal extent of the defect were determined on coronal T1 TSE images at both time points. At the corresponding axial level, the defect outline was assessed on AX PD SPAIR images at both time points to define the base of the defect. MRI acquisition parameters and patient positioning followed the standard institutional knee MRI protocol used in routine postoperative assessment. For calculation purposes, the defect was approximated as a triangular prism, and volume was estimated using a simplified geometric model based on the measured defect base and longitudinal extent. Volume was expressed in cubic millimeters (mm^3^) (Figure 1 and Figure 2).

Measurements were performed at 4 weeks and 12 months after surgery by two independent evaluators, in consultation with radiology specialists, both of whom were blinded to the patient-reported outcome scores. For each parameter, the final value was defined as the mean of the two measurements. Formal interobserver and intraobserver reliability analyses were not performed. Based on these values, the percentage of bone defect regeneration during the 1-year postoperative period was calculated as follows:

Regeneration (%) = [(defect volume at 4 weeks − defect volume at 12 months)/defect volume at 4 weeks] × 100

### 2.4. Clinical Evaluation

Clinical outcomes were assessed at 12 months postoperatively using the original versions of the International Knee Documentation Committee (IKDC) subjective knee form and the Donor Site Morbidity Questionnaire (DSMQ) [14,15]. The questionnaires were completed during follow-up visits together with the attending orthopedic surgeon. The IKDC is a standardized patient-reported measure of symptoms and function, scored from 0 to 100, with higher scores indicating better knee function. The DSMQ is a patient-reported instrument designed to assess donor-site morbidity after ACL reconstruction with a patellar tendon autograft, with higher scores indicating fewer donor-site-related complaints.

### 2.5. Statistical Analysis

Statistical analyses were performed using JASP software (JASP Team, Amsterdam, The Netherlands), version 0.95.4.0. The normality of data distribution was evaluated using the Shapiro–Wilk test. For comparison between independent groups and subgroups, Student’s *t*-test was utilized for normally distributed data, whereas the Mann–Whitney U test was applied when the assumption of normality was violated. Comparisons between dependent continuous measurements were performed using the paired Student’s *t*-test, with Cohen’s d calculated to determine the corresponding effect size. A formal a priori sample size calculation was not conducted due to the limited nature of our consecutive patient cohort. Instead, a post hoc power analysis was performed using G*Power, version 3.1.9.7 (Heinrich Heine University Düsseldorf, Düsseldorf, Germany). Based on our sample size and assuming a medium effect size (d = 0.5), the analysis estimated a critical t-value of approximately 1.7, and the achieved power (1-β) was 0.85. Associations between MRI-derived parameters and patient-reported outcomes were assessed using correlation analysis. Spearman’s rank correlation coefficient was used to examine correlations between quantitative variables, and the corresponding correlation coefficients were interpreted as indicators of effect size according to Cohen’s criteria. To provide a robust assessment of these effect sizes, Fisher’s z transformation (with standard error adjusted for rank-based data) was applied to calculate the 95% confidence intervals (95% CI) for each correlation coefficient. *p*-values < 0.05 were considered statistically significant.

### 2.6. Ethics

The study was approved by the Ethics Committee of the University Clinical Center of Vojvodina (16 September 2022; No. 00-161). Written informed consent was obtained from all participants prior to inclusion in the study.

No generative artificial intelligence (GenAI) was used in the preparation of this manuscript beyond superficial language editing (grammar, spelling, punctuation, and formatting). All scientific content was developed, reviewed, and approved by the authors.

## 3. Results

### 3.1. Baseline Characteristics

A total of 30 consecutive eligible patients with complete data were included in the analysis. The baseline demographic and clinical characteristics of the study cohort are presented in Table 1.

### 3.2. MRI-Based Bone Defect Volume and Regeneration

At 4 weeks after surgery, the mean donor-site bone defect volume was 2602.4 ± 684.7 mm^3^ in the patella and 2993.9 ± 714.3 mm^3^ in the tibia. At 12 months, defect volume decreased at both sites, to 628.0 ± 279.7 mm^3^ in the patella and 980.8 ± 488.2 mm^3^ in the tibia. Tibial defects were significantly larger than patellar defects at both time points (*p* = 0.026 at 4 weeks and *p* = 0.0012 at 12 months). The corresponding effect sizes indicated a moderate difference at 4 weeks (Cohen’s d = 0.56) and a large difference at 12 months (Cohen’s d = 0.88), suggesting a stronger separation between donor sites at the later time point. The mean regeneration percentage between 4 weeks and 12 months was 74.84 ± 11.51% for the patella and 67.23 ± 15.10% for the tibia, with significantly greater regeneration in the patella (*p* = 0.0264). The corresponding effect size was moderate (Cohen’s d = 0.57) (Table 2).

In secondary exploratory analyses, no significant sex-related differences were observed in bone defect volume, regeneration percentage, or IKDC and ACL-DSMQ scores. According to the side of surgery, patellar defect volume at 12 months was greater in the right knee (*p* = 0.0402), while patellar regeneration was lower on the right side (*p* = 0.0232); no significant side-related differences were found for tibial defect parameters. These side-related findings should be interpreted cautiously, given the exploratory nature of the analysis and the possibility of chance findings.

### 3.3. Clinical Outcomes and Correlations with MRI Parameters

At 12 months, the mean IKDC score was 67.1 ± 13.2, and the mean ACL-DSMQ score was 78.6 ± 18.6. A strong positive correlation was observed between IKDC and ACL-DSMQ scores (Spearman’s ρ = 0.689, *p* < 0.001) (Table 3).

The percentage of defect regeneration in both the patella and tibia was not significantly associated with either IKDC or ACL-DSMQ scores. In contrast, larger patellar defect volume at 4 weeks showed moderate negative correlations with both IKDC (ρ = −0.459, 95% CI −0.739 to −0.075; *p* = 0.0107) and ACL-DSMQ (ρ = −0.459, 95% CI −0.759 to −0.072; *p* = 0.0107). Patellar defect volume at 12 months was not associated with IKDC, but showed a weak negative correlation with ACL-DSMQ (ρ = −0.362, 95% CI −0.650 to −0.006; *p* = 0.0495). No significant correlations were found between tibial defect parameters and either patient-reported outcome measure. Overall, the observed correlations between MRI-derived parameters and patient-reported outcomes were weak to moderate, with the most consistent findings involving patellar defect volume rather than regeneration percentage. Age, height, body weight, and BMI were not significantly associated with defect volume, regeneration percentage, IKDC, or ACL-DSMQ scores (all *p* > 0.05).

## 4. Discussion

The main findings of the present study were that tibial donor-site bone defects were larger than patellar defects at both 4 weeks and 12 months after BTB ACL reconstruction, whereas the patella showed a greater regeneration percentage over time. In addition, the regeneration percentage itself was not associated with patient-reported outcomes, while larger patellar defect volume at 4 weeks was associated with worse subjective outcomes at 12 months. Patellar defect volume at 12 months was also negatively associated with donor-site morbidity, whereas tibial defect parameters showed no significant correlations with clinical scores.

These findings add to the current understanding of donor-site healing after BTB graft harvest. BTB autografts remain widely used because of their favorable biomechanical characteristics and reliable fixation, but donor-site morbidity continues to be one of their main limitations [1,4,16]. Previous studies have shown that donor-site symptoms, particularly anterior knee pain and kneeling difficulty, remain clinically relevant after BTB ACL reconstruction [2,4,6,15,17,18]. In the present study, tibial defects remained larger than patellar defects at both evaluated time points, while the patella showed a greater regeneration percentage. This pattern indicates site-specific differences in biological healing after graft harvest, although the exact mechanisms remain insufficiently defined. Alternative explanations, including differences in postoperative loading patterns, rehabilitation-related factors, and individual biological healing responses, should also be considered.

Our findings should also be considered in light of studies that investigated donor-site defect filling. Bone filling and biologic augmentation techniques have been associated with improved donor-site healing and reduced morbidity after BTB graft harvest [8,9,19,20]. These reports support the concept that donor-site management may influence both structural healing and patient symptoms. In our cohort, defects were left unfilled, and measurable donor-site defects remained present at 12 months. The fact that tibial defects remained larger while patellar defects showed greater relative regeneration suggests that donor-site healing is not uniform and may differ according to anatomical location.

An important observation in the present study was that regeneration percentage was not significantly associated with either IKDC or ACL-DSMQ scores. This suggests that the degree of radiological defect filling over time does not necessarily parallel the patient’s subjective recovery. A similar dissociation between structural findings and clinical symptoms has been described previously in the donor-site morbidity literature [21]. Our results also showed that larger patellar defect volume at 4 weeks was associated with worse IKDC and ACL-DSMQ scores at 12 months, indicating that the early extent of donor-site injury may be more clinically relevant than the final percentage of regeneration itself. However, these associations should be interpreted cautiously, given the modest sample size and the observational cohort design of the study.

The association between patellar defect parameters and patient-reported outcomes is clinically plausible. The mean IKDC score in our cohort indicated only moderate subjective knee function at 12 months, suggesting that recovery remained incomplete in a proportion of patients despite surgical treatment. Tsuda et al. reported that residual donor-site defects may contribute to anterior knee symptoms, supporting the interpretation of our findings [22]. In our study, patellar defect volume at 12 months was not associated with IKDC, but showed a weak negative correlation with ACL-DSMQ, suggesting that persistent patellar donor-site structural changes are more closely related to local donor-site complaints than to overall knee function. In contrast, tibial defect parameters were not significantly associated with either IKDC or ACL-DSMQ, which indicates that tibial defects, although larger in absolute terms, are less relevant to subjective morbidity than patellar defects.

An additional strength of the present study is that it prospectively evaluated donor-site bone defects at two predefined postoperative time points and related MRI-based findings to both a general knee function score and a donor-site-specific morbidity instrument. To our knowledge, and based on the literature reviewed for this study, we found no previous report that quantitatively evaluated both patellar and tibial donor-site bone defects on MRI and correlated these findings with patient-reported outcomes after BTB ACL reconstruction. In this regard, the present study extends the available literature, which has mainly focused on donor-site symptoms, technical modifications, defect filling, or tendon-related healing rather than on quantitative MRI assessment of both osseous harvest sites [4,8,9,15,17,19,20,22,23].

The present findings also have clinical implications. In particular, the observed association between larger early patellar defects and worse subjective outcomes suggests that donor-site structural changes may be clinically relevant in the postoperative evaluation of patients after BTB anterior cruciate ligament reconstruction. These findings may be useful in postoperative patient counseling and in the interpretation of persistent donor-site symptoms, particularly anterior knee complaints after BTB graft harvest. In patients at increased risk of donor-site morbidity, particularly those for whom kneeling ability is important, surgeons may consider techniques intended to reduce harvest-site morbidity, such as donor-site defect filling or biologic augmentation [8,9,18,19]. At the same time, these results should not be interpreted as an argument against BTB autografts, since this graft remains a reliable option with well-established biomechanical advantages and good long-term outcomes [1,5,24,25,26,27]. Rather, the present findings support more individualized patient counseling and further refinement of donor-site management. At present, these findings do not support routine use of MRI-based defect volumetry in all postoperative patients, but they may be relevant in selected cases with persistent donor-site symptoms.

Limitations of this study include a relatively small single-center sample, simplified geometric estimation of bone defect volume, lack of formal interobserver and intraobserver reliability analysis, and the absence of multivariable adjustment. In addition, follow-up was limited to 12 months and may not fully reflect longer-term donor-site structural and clinical outcomes.

Future studies with larger cohorts, standardized imaging protocols, reliability testing, and more advanced volumetric methods are needed to clarify the clinical significance of donor-site bone healing and to determine whether modifications in harvest-site management can improve patient outcomes.

## 5. Conclusions

After BTB anterior cruciate ligament reconstruction, tibial donor-site bone defects were larger than patellar defects, whereas the patella showed greater regeneration over time. Regeneration percentage was not associated with IKDC or ACL-DSMQ scores; however, larger patellar defect volume at 4 weeks was associated with worse subjective outcomes at 12 months. These findings suggest that while larger early patellar donor-site defects may be associated with worse subjective outcomes, their exact clinical relevance remains uncertain. Consequently, the prognostic and clinical value of MRI-based defect volumetry should be confirmed in larger, methodologically standardized studies.

## Figures and Tables

**Table 1:** Baseline demographic and clinical characteristics of the study cohort.

Number of patients, n	30
Sex, n (%)	Male 26 (86.7%); Female 4 (13.3%)
Side of surgery, n (%)	Right 15 (50.0%); Left 15 (50.0%)
Age (years), mean ± SD	32.7 ± 8.2
Height (m), mean ± SD	1.8 ± 0.1072
Body weight (kg), mean ± SD	82.9 ± 13.9
BMI (kg/m^2^), mean ± SD	26.1 ± 3.1

**Table 2:** MRI-based donor-site bone defect volume at 4 weeks and 12 months and regeneration percentage after bone–patellar tendon–bone ACL reconstruction.

Donor Site	Defect Volume at 4 Weeks (mm^3^), Mean ± SD	Defect Volume at 12 Months (mm^3^), Mean ± SD	Regeneration (%), Mean ± SD
Patella	2602.4 ± 684.7	628.0 ± 279.7	74.8 ± 11.5
Tibia	2993.9 ± 714.3	980.8 ± 488.2	67.2 ± 15.1
*p* value	0.026	0.0012	0.0264

Note: *p*-values represent comparisons between patellar and tibial donor-site defects at each time point and for regeneration percentage.

**Table 3:** Correlations between MRI-derived donor-site bone defect parameters and patient-reported outcomes at 12 months.

MRI Parameter	IKDC (ρ)	*p*	ACL-DSMQ (ρ)	*p*
Patellar defect at 4 weeks (mm^3^)	−0.459	0.0107	−0.459	0.0107
Patellar defect at 12 months (mm^3^)	0.053	0.782	−0.362	0.0495
Tibial defect at 4 weeks (mm^3^)	−0.090	0.636	−0.070	0.713
Tibial defect at 12 months (mm^3^)	0.184	0.331	0.120	0.528
Patellar regeneration (%)	0.268	0.151	0.150	0.430
Tibial regeneration (%)	−0.228	0.225	−0.189	0.317

Note: Values are presented as Spearman’s rank correlation coefficients (ρ). IKDC, International Knee Documentation Committee; ACL-DSMQ, Anterior Cruciate Ligament Donor Site Morbidity Questionnaire.

## Data Availability

The data presented in this study are available on reasonable request from the corresponding author.
